# Ependymal alterations in sudden intrauterine unexplained death and sudden infant death syndrome: possible primary consequence of prenatal exposure to cigarette smoking

**DOI:** 10.1186/1749-8104-5-17

**Published:** 2010-07-19

**Authors:** Anna M Lavezzi, Melissa F Corna, Luigi Matturri

**Affiliations:** 1 'Lino Rossi' Research Center, Department of Surgical, Reconstructive and Diagnostic Sciences, University of Milan, Via della Commenda 19, 20122 Milan, Italy

## Abstract

**Background:**

The ependyma, the lining providing a protective barrier and filtration system separating brain parenchyma from cerebrospinal fluid, is still inadequately understood in humans. In this study we aimed to define, by morphological and immunohistochemical methods, the sequence of developmental steps of the human ependyma in the brainstem (ventricular ependyma) and thoracic spinal cord (central canal ependyma) of a large sample of fetal and infant death victims, aged from 17 gestational weeks to 8 postnatal months. Additionally, we investigated a possible link between alterations of this structure, sudden unexplained fetal and infant death and maternal smoking.

**Results:**

Our results demonstrate that in early fetal life the human ependyma shows a pseudostratified cytoarchitecture including many tanycytes and ciliated cells together with numerous apoptotic and reactive astrocytes in the subependymal layer. The ependyma is fully differentiated, with a monolayer of uniform cells, after 32 to 34 gestational weeks. We observed a wide spectrum of ependymal pathological changes in sudden death victims, such as desquamation, clusters of ependymal cells in the subventricular zone, radial glial cells, and the unusual presence of neurons within and over the ependymal lining. These alterations were significantly related to maternal smoking in pregnancy.

**Conclusions:**

We conclude that in smoking mothers, nicotine and its derivatives easily reach the cerebrospinal fluid in the fetus, immediately causing ependymal damage. Consequently, we suggest that the ependyma should be examined in-depth first in victims of sudden fetal or infant death with mothers who smoke.

## Background

The ependyma (EP), the lining of the cerebral ventricles and central canal of the spinal cord in mammalian species, consists of a single uninterrupted layer of a type of glial cells (the ependymal cells). Their apical surfaces are provided with cilia, which circulate cerebrospinal fluid (CSF) around the central nervous system, and with microvilli, which absorb CSF. Interposed among these mural cells are specialized forms of ependymal cells, the 'tanycytes', characterized by basal processes directed into the subjacent neuropil.

Although the EP has been the subject of numerous studies in animals over the years [1-6], there is still little information about this layer in humans, and compared to other central nervous system structures, the pathological development of the human EP has been largely ignored. Even in our previous reports showing abnormalities of nuclei and/or structures of the brainstem and cerebellum in sudden intrauterine unexplained death (SIUD) and sudden infant death syndrome (SIDS), the EP was not studied in depth [7-12]. Therefore, the aim of the present study was to focus on this important structure and, in particular, to evaluate if the EP also shows morphofunctional alterations in victims of unexplained death.

We first investigated the developmental patterns of the EP in a wide sample of subjects aged from 17 gestational weeks to 10 months of life who had died of known and unknown causes, and then we evaluated the presence of morphofunctional disorders of this lining in SIUD/SIDS. The study protocol included, in all cases: morphological examination of histological sections of the EP in the medulla oblongata and spinal cord; immunohistochemical evaluation of apoptosis in this lining (by the TUNEL method (deoxynucleotidyl transferase (TdT)-mediated dUTP nick end labeling)) and of the reactive glia (by glial fibrillary acidic protein (GFAP) expression). Finally, in view of the observations in our previous studies of a significantly increased incidence of structural and/or functional alterations of the central autonomic nervous system in victims of unexplained perinatal and infant death with mothers who smoke ('smoker mothers') [13-15], we evaluated whether prenatal absorption of nicotine could interfere with the maturational processes of the EP.

## Materials and methods

### Study subjects

The study included three cohorts of victims: a SIDS group, a SIUD group, and a control group (for a total of 78 cases).

#### SIDS and SIUD victims

The SIDS cases included 30 infants, 12 females and 18 males, aged from 1 to 8 postnatal months (median age, 3.7 months). The SIUD cases included 24 unexplained ante-partum deaths, 11 females and 13 males, aged 25 to 41 gestational weeks (median age, 38 weeks). This was a selected set of cases, sent to our research center over a 3-year period (2006 to 2009), and diagnosed according to the application of the 2006 guidelines stipulated by Italian law n.31 'Regulations for Diagnostic Post Mortem Investigation in Victims of SIDS and Unexpected Fetal Death'. This law decrees that all infants suspected of SIDS who died suddenly in Italian regions within the first year of age, as well as all fetuses who died after the 25th week of gestation without any apparent cause, must undergo an in-depth anatomopathological examination, particularly of the autonomic nervous system.

For every case, a complete clinical history was collected. Additionally, mothers were asked to complete a questionnaire on their smoking habit, detailing the number of cigarettes smoked before, during and after pregnancy. Of the 54 SIDS/SIUD mothers, 22 (40%) were active smokers before and during the pregnancy, smoking more than 3 cigarettes per day. The remaining 32 mothers (60%) admitted no history of cigarette smoking.

#### Controls

This group included 24 victims of sudden death (12 infants (3 females, 9 males) aged from 1 to 8 postnatal months (median age, 3.4 months), and 12 fetuses (6 females, 6 males) aged 17 to 40 gestational weeks (median age, 35 weeks)) for whom a complete autopsy and clinical history analysis established a precise cause of death. The related infant death diagnoses were: congenital heart disease (n = 5), severe bronchopneumonia (n = 3), myocarditis (n = 1), pulmonary dysplasia (n = 2), and mucopolysaccharidosis type I (n = 1). Specific diagnoses among the fetal deaths included: chorioamnionitis (n = 7) and congenital heart disease (n = 5). Of the 24 mothers of the control group, 7 (29%) reported a smoking habit. The remaining 16 mothers (71%) were non-smokers.

All the victims of the study were subjected to a complete autopsy, including examination of the placental disk, umbilical cord and membranes in fetal deaths. In all cases an in-depth histological examination of the autonomic nervous system was made, in accordance with the protocol of the 'Lino Rossi Research Center for the Study and Prevention of Unexpected Perinatal Death and Sudden Infant Death Syndrome' of Milan University [16,17].

In particular, after fixation in 10% phosphate-buffered formalin, the brainstem and spinal cord were processed and embedded in paraffin. Transverse serial sections of the midbrain, pons, medulla oblongata and thoracic spinal cord were made at intervals of 60 μm. For each level, ten 5-μm sections were obtained, three of which were stained for histological examination using hematoxylin-eosin, Klüver-Barrera stains and Bielchowsky's silver impregnation technique; additional sections at each level were subjected to immunohistochemistry for the study of apoptosis and gliosis. The remaining sections were saved and stained as deemed necessary for further investigations.

The in-depth examination of the EP, the target of this study, was performed in all cases on the surface of the fourth ventricle at the same level of the medulla oblongata (on the histological sections corresponding to the obex) and on the lining of the central canal at levels T2-T3 of the thoracic spinal cord. The selected sections also allow easy analysis of further important structures (the dorsal motor vagus, tractus solitarius, ambiguous, pre-Bötzinger, inferior olivary and arcuate nuclei in the medulla oblongata and intermediolateral nucleus in the spinal cord). In addition, we analyzed the locus coeruleus and the parabrachial/Kölliker-Fuse complex in the rostral pons/caudal midbrain, the facial/parafacial complex and the superior olivary complex in the caudal pons.

### Immunohistochemistry

#### Glial fibrillary acidic protein immunostaining

Sections were deparaffinized and washed in phosphate-buffered saline. After blocking endogenous peroxidase with 3% H_2_O_2_, the slides were pretreated in a microwave oven using a citrate solution (pH = 6). Then the sections were incubated overnight with primary monoclonal antibody NCL-GFAP-GA5 (anti-GFAP, Novocastra, Newcastle Tyne, UK) at a dilution of 1:300. Immunohistochemical staining was performed with the peroxidase-antiperoxidase method and the avidin-biotin complex technique (ABC Kit, Vectastain, Vector Laboratories Inc., Burlingame, CA, USA). Diaminobenzidine (DAB, Vector Laboratories Inc.) was used as chromogen substrate and counterstained with light hematoxylin. Negative controls of the same tissue were done using phosphate-buffered saline instead of primary antibody.

To evaluate the GFAP density (expressed as the number of reactive astrocytes per mm^2^), all the immunopositive reactive astrocyte cells were counted in transverse sections, using an optical microscope at 40 × magnification. The morphologic criteria to identify reactive astrocytes were a large vesicular nucleus and conspicuous fibrillary processes extending into the surrounding neuropil.

#### Apoptosis immunostaining (TUNEL method)

The sections were deparaffinized and incubated with 20 μg/ml proteinase K (Sigma; St. Louis, MO, USA). After blocking the endogenous peroxidase with 3% hydrogen peroxide, TdT (0.3 U/ml) was used to incorporate digoxigenin-conjugated deoxyuridine (dUTP 0.01 mM/ml) into the ends of DNA fragments. The TUNEL signal was then detected by an anti-digoxigenin antibody conjugated with peroxidase (Apoptag Peroxidase *In Situ *Apoptosis Detection kit, Oncor, Gaithersburg, MD, USA). Counterstaining was performed by immersing the slides in methyl green for 10 minutes.

### Statistical analysis

The statistical significance of direct comparison between the groups of victims was determined using analysis of variance (ANOVA). Statistical calculations were carried out on a personal computer with SPSS statistical software (version 11.0; SPSS Inc., Chicago, IL, USA). The selected threshold level for statistical significance was *P *< 0.05.

## Results

### Morphological stages of human EP development

**Figure 1:**
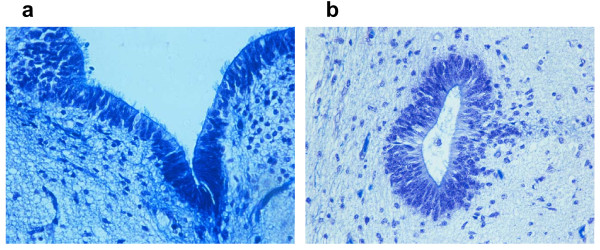
**Thick, pseudostratified ependymal layer of a 17-week human fetus. (a) **In the lining of the fourth ventricle (histological section of brainstem); **(b) **in the lining of the central canal (histological section of thoracic spinal cord). Klüver-Barrera stain; magnification, 20×.

At the earliest stages, in fetuses aged 17 to 19 gestational weeks of the control group, the EP consisted of a thick stratified lining of small round cells with an uninterrupted ciliated layer that lines the fourth ventricle of the brainstem and the central canal of the spinal cord (Figure 1a,b).

**Figure 2:**
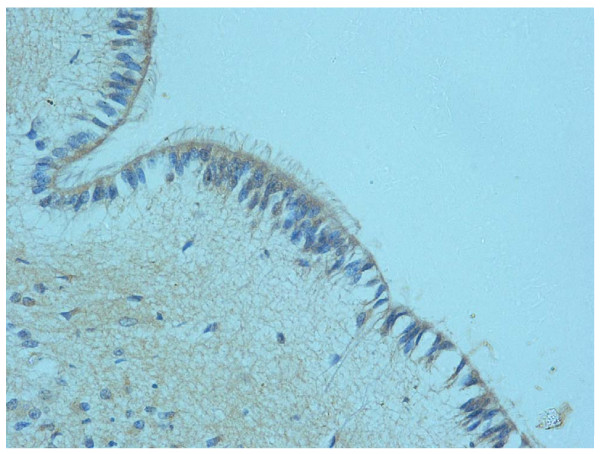
**Clusters of cilia in the monostratified ventricular ependyma of a fetus aged 28 gestational weeks. **Klüver-Barrera stain; magnification, 40×.

**Figure 3:**
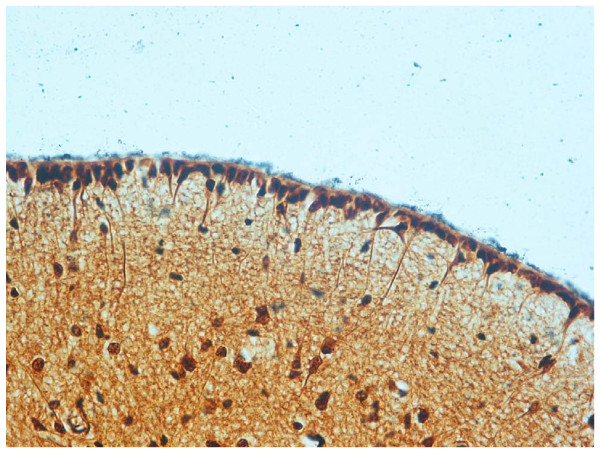
**Ventricular ependyma with many tanycytes in a 30-week fetus. **Bielchowsky's silver impregnation technique; magnification, 40×.

**Figure 4:**
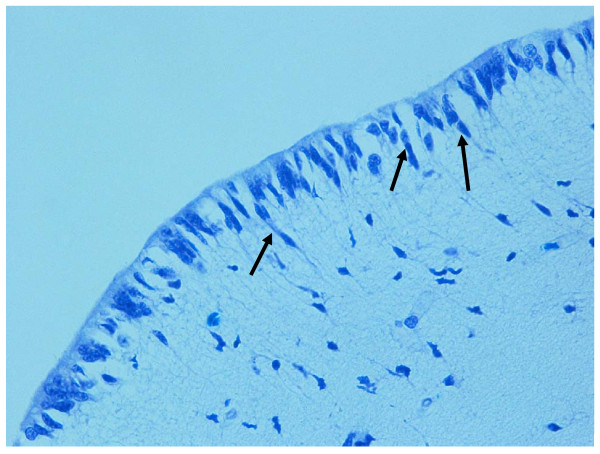
**Lateral ventricular surface with many tanycytes arranged in radial glial formations (arrows) in a fetus aged 30 gestational weeks. **Klüver-Barrera stain; magnification, 40×.

**Figure 5:**
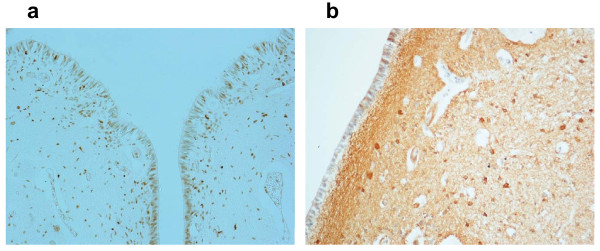
**Floor of fourth ventricle of a fetus aged 30 gestational weeks showing high expression of (a) apoptosis and (b) GFAP immunopositivity in the ependyma and subependymal layer. (**Klüver-Barrera stain; magnification, 20×.

From 20 to 30 gestational weeks, the ventricular EP gradually becomes thinner until it appears as a single layer of cuboidal/columnar neuronal cells arranged in a disorderly manner. Clusters of well differentiated cilia cover the surface of many external cells (Figure 2). Most ependymal cells (tanycytes) have radially directed basal processes that extend for a variable distance into the subjacent neuropil, defined as the 'subventricular zone' (SVZ), where they frequently enwrap blood vessels or terminate on neurons (Figure 3). Tanycytes are often arranged in rows that terminate in the EP and share features with radial glia (Figure 4). The ependymal cells and the subependymal layer show strong GFAP and TUNEL immunoreactivity (Figure 5).

The ependymal development is complete by 32 to 34 weeks of gestation and maintains the same cytoarchitecture in the first months of life. The mature ventricular EP is a single layer of cuboidal to columnar cells with a fairly round nucleus, fine stippled chromatin and inconspicuous nucleolus. Ciliated ependymal cells are decreased in number or not fully observable. Chains of radial glial cells are a rare finding. The SVZ is thinner with few reactive astrocytes. There are few apoptotic cells. The EP lining the central canal in the spinal cord differs from the ventricular EP only inasmuch as it preserves a pseudostratified structure consisting of cuboidal or columnar cells.

### Pathologic human EP development in SIDS/SIUD

The SIDS and SIUD cases showed a significantly high incidence of histological/immunohistochemical alterations mostly affecting the ventricular EP compared with age-matched controls. Overall, in 29 of the 54 victims of sudden death (54%) and in 4 of the 24 subjects belonging to control group (17%) we observed EP modifications (*P *< 0.01).

**Figure 6:**
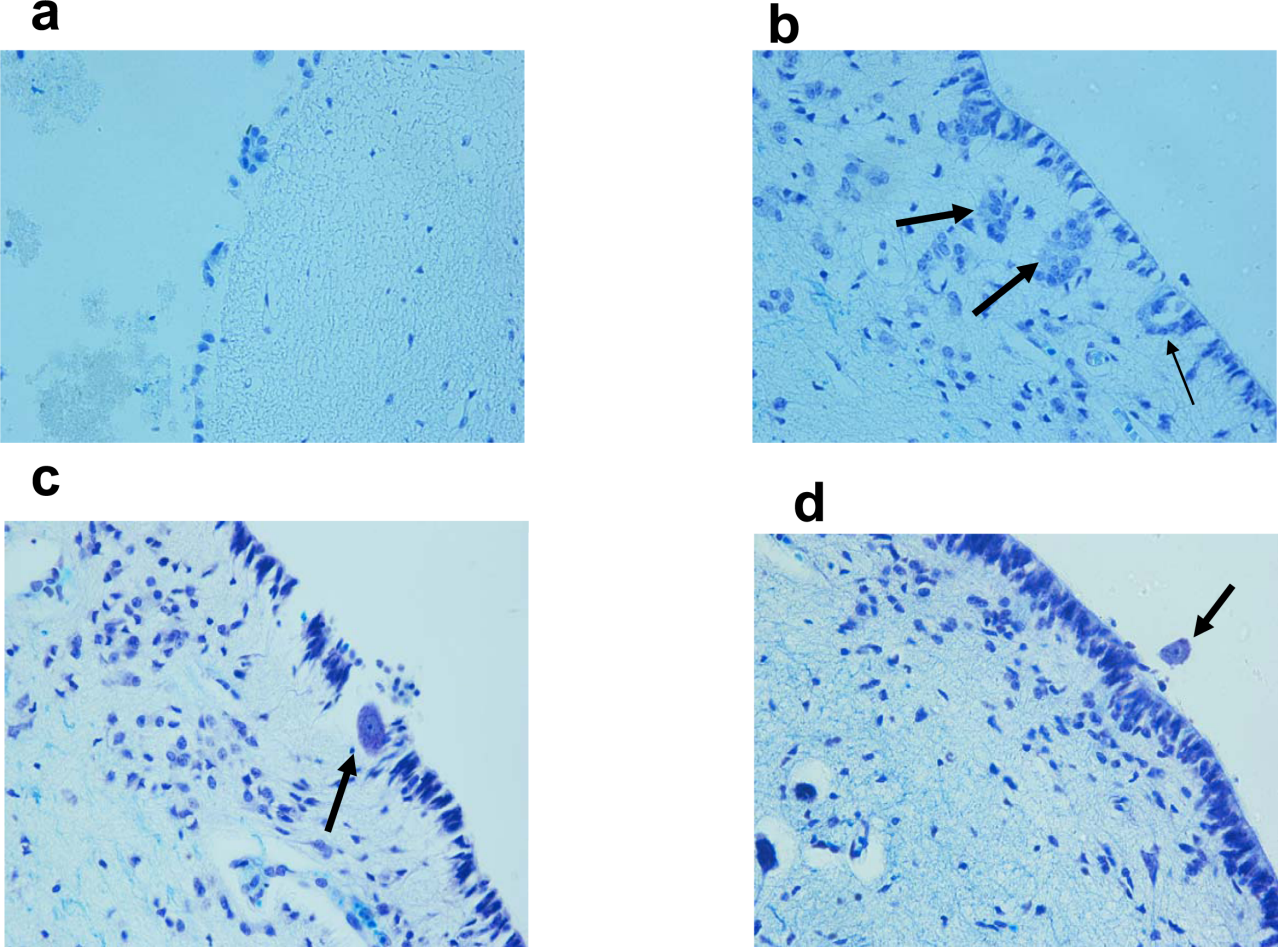
**Pathological ependyma in sudden fetal and infant death victims. (a) **Wide area of desquamation in a SIUD case aged 38 gestational weeks. **(b) **Ependymal invaginations (thin arrow) and nodules (thick arrows) in a 3-month-old infant who died of SIDS. **(c,d) **Neuronal heterotopia. Isolated neurons within (c) and over (d) the ependymal lining (arrows) in a SIDS victim aged 2 months. Klüver-Barrera stain; magnification, 40×.

In early SIUD cases (aged 25 to 30 gestational weeks) the main defects were: tanycytes only rarely present; atrophy of the cilia; and a low number of apoptotic cells and reactive astrocytes. In late SIUD cases (from 38 to 41 gestational weeks) we frequently observed: micro- or macro-areas of EP desquamation and/or discontinuity on the ventricular surface (Figure 6a); marked gliosis, validated by GFAP immunopositivity, in the SVZ; a high apoptotic cell density in the EP and SVZ; and an increased number of immunopositive glial cells compared to findings in controls.

In SIDS the main alterations were: clusters and/or nodules of ependymal cells in the SVZ derived from diverticuli of the EP surface penetrating into the subjacent brainstem parenchyma (Figure 6b); ventricular membrane desquamation/denudation; a high number of tanycytes, indicative of delayed EP maturation, and radial glia formations; the unusual presence of single neurons positioned within or over the ependymal monolayer and in contact with the ventricular lumen (neuronal heterotopia) (Figure 6c,d); reactive gliosis overgrowing the EP edge; thickening of the SVZ with glial proliferation and vacuolation, usually below areas of EP cell deprivation. Discontinuities and/or invaginations were also frequently observed in the EP of the central canal in the spinal cord of SIDS victims.

In the control group the EP structure was rarely altered. Table 1 shows the distribution of the main EP defects in victims of sudden death and in controls; it must be remembered that individual victims may display any combination of these pathological findings. The most frequent association was EP loss and the presence of glial chains in adjacent areas.

**Table 1:** Distribution of the main ventricular and spinal cord ependymal alterations in SIUD/SIDS and controls

Ependymal alteration	SIUD victims (n = 24)	Control fetal death victims (n = 12)	SIDS victims (n = 30)	Control infant deaths (n = 12)
Morphological features				
EP desquamation	9	1	19	3
EP invaginations and nodules in subependymal layer	3	1	16	1
Altered number of EP cells with cilia	12^a^	2	14^b^	0
Altered number of tanycytes	17^a^	0	21^b^	2
Altered number of radial glia chains	14^a^	0	12^b^	0
Neuronal heterotopia	0	0	11	0
Immunohistochemical features				
High GFAP-positivity	20	0	18	2
High apoptotic positivity	18	0	13	0

Individual victims may display any combination of these alterations. ^a^Decreased number; ^b^increased number.

In most SIDS/SIUD victims with developmental abnormalities of the EP, we also observed morphological alterations of different brainstem and spinal cord structures (namely hypoplasia/agenesis of the arcuate nucleus, pre-Bötzinger nucleus, inferior olivary nucleus, serotonergic raphé nuclei, parafacial nucleus in the medulla oblongata/pons, and intermediolateral nucleus in the thoracic spinal cord). The most frequent associations were between EP alterations and hypodevelopment of the serotonergic raphé nuclei and of the arcuate nucleus in SIDS victims and hypoplasia of the pontine parafacial nucleus in sudden fetal deaths. In the control group, hypoplasia of the arcuate nucleus was frequent.

### Correlation of findings with smoking exposure

We observed a significant relationship between maternal smoking, sudden unexplained death and alterations of the EP. In fact, 15 of the 22 sudden death victims with a smoker mother (68%) showed developmental alterations of the EP. In the control group three of the four cases with EP changes had a smoker mother, confirming the association between smoking and EP alterations.

## Discussion

Even if the EP attracted the attention of researchers long ago [18-20], few authors have made in-depth studies of the development, morphological features and pathological aspects of this structure in humans. Among these, Del Bigio [21], in a recent review, summarizes the current knowledge of the biology and pathology of the EP in mammalian brains. In particular, the author underlines that the EP has potential barrier functions at the brain-CSF interface, including the upregulation of 'protective' proteins that might prevent re-entry of harmful metabolites from the CSF back into the brain. The presence of motile cilia is important to carry out this role.

Narita *et al*. [22] demonstrated that primary cilia in EP may function as chemosensors, thus playing an important role in regulating the homeostasis of the central nervous system. The EP also has transport systems and distinct enzymes that are able to remove endogenous and exogenous toxins from the nervous system [23-25]. The activity of tanycytes, the nonciliated ependymal cells characterized by basal processes penetrating into the brain parenchyma and frequently reaching small blood vessels, could be included in this context [26]. The connections made by the tanycytes from the CSF to neural capillaries raise the possibility that they may play some role in sampling the biochemical constituents of both compartments, thereby allowing the uptake and transport of substances into and out of the CSF, so influencing neuronal activity. The participation of tanycytes in chemoreception is, however, a function limited to fetal life. Tanycytes, in fact, represent a transitional cell during human fetal development and not a distinct population. They gradually mature into common ependymal cells following loss of the basal processes [27].

In this study, direct investigation of the ventricular and spinal cord EP in a wide set of fetal and infant deaths (78 cases), victims of both unexplained and explained death, firstly allowed us to delineate the dynamic sequence of morphological and biological steps occurring in human EP development from gestational week 17. We observed that the ependymal lining is fully differentiated by approximately 32 to 34 weeks of gestation, which is several weeks later than reported by Del Bigio [21] and a few weeks before the estimation by Spassky *et al*. [28] that EP maturation occurs during the first postnatal week. In early fetal life ventricular EP showed a pseudostratified cytoarchitecture, including many tanycytes and ciliated cells together with strong GFAP and TUNEL immunoreactivity, whereas in late fetal life and in the first postnatal months it is formed by a single layer of cuboidal or columnar cells that are rarely immunopositive.

Additionally, this study identified a wide spectrum of pathological changes of the EP in a large proportion of sudden death victims. These prevalently included: desquamation or denudation; glial clusters and vacuolation in the SVZ beneath a discontinuous EP; chains of radial glial cells, particularly in SIDS victims; reactive gliosis and heterotopic neurons inside and over the EP edge.

Glial nodules rise from sequestration of EP diverticuli penetrating into the parenchyma. It is not clear what role these invaginations of the EP might have, and this topic merits further investigation. The subependymal vacuolation could be related to ependymal cell loss. The discontinuous lining may not be able to perform its function in regulating the transport of fluid, ions and small molecules between the CSF and the cerebral parenchyma, and thus may contribute to subependymal edema and the formation of vacuoles that could be interpreted as segregation of the excessive fluid.

The so-called 'radial glial cells' are generally recognized as elongated astrocytes with long processes arranged in small chains that run across the neuropil perpendicularly to the EP line. Experimental studies in mice demonstrated that a subpopulation of radial glia serves as progenitors of new ependymal cells during development [29]. So, we can interpret the chains of radial glial cells associated with EP disruptions observed in SIDS victims as an attempt to reconstruct an intact border between the brain parenchyma and the ventricular cavity.

The heterotopic presence of single intraependymal and supraependymal neurons observed in SIDS is more difficult to understand. It has long been well known that some neuronal elements send their processes to the CSF. Using silver impregnation methods, Parent [30] demonstrated that several serotonergic axons of neurons belonging to the raphé nuclei terminate in the ventricular surface of the EP entering the CSF. Similarly, long basal processes from neurons of the arcuate nucleus in mammals are able to run into the subependymal neuropil reaching the ventricle [31]. These neurons represent a component of the so-called 'CSF-contacting neuronal system' [32], which is an important nonsynaptic signal transmission in the brain. Three peculiar types of CSF-contacting neurons have been recognized: intraependymal neurons, which line the walls of ventricles and of the central canal of the spinal cord; supraependymal cells, which are subjacent to the ependyma; and distal CSF-contacting neurons, whose body is in the parenchyma of the brain and whose processes extend into the CSF in the ventricle system [33].

The same brainstem nucleus can include neurons with intraventricular processes modulating their activity according to the composition of the ventricular CSF, as well as neurons that work according to the composition of the intercellular fluid. The connection between the two types of neurons may lead to the detection of differences between the two fluids, so coordinating a nonsynaptic transmission. Nevertheless, we cannot exclude that single cells belonging to the first group of neurons may dedifferentiate, migrate outside their original localization and directly establish contact with the CSF of the ventricle. These heterotopic neuronal cells could, at last, represent newly generated neurons in the SVZ. In the mouse brain, in fact, the SVZ represents a neurogenic stem cell niche that controls neurogenesis and gliogenesis [34-36]. In this regard, a subpopulation of SVZ astrocytes has been identified as comprising stem cells that are able to generate neuroblasts, although it is not yet known exactly how intercellular communication regulates SVZ neurogenesis [35].

In any case, the EP lesions are generally irreversible and, regardless of the type of alteration, the damaged EP may not be able to perform its primary function as a protective barrier between the brain and the CSF, selectively removing noxious substances from the nervous system [23-25]. We suggest that many of the EP alterations that we observed in this study are indicative of prenatal injury that could induce dysfunctions in the control of vital functions, like the developmental abnormalities of other nuclei and/or structures of the brainstem and cerebellum that we have reported in previous work [13-15].

Experimental studies have demonstrated that in pathological situations, such as viral infections, destruction of the EP is the main outcome [3,37,38]. Chronic brain hypoxia/ischemia in rats rapidly allows EP cells to reacquire a radial glia phenotype in SVZ [39-41]. Rothstein and Levison [42] confirmed that in conditions of cerebral hypoxia the EP and SVZ cells are most vulnerable and easily undergo extensive damage.

In our view cigarette smoke is the main factor involved in human EP alterations in unexplained fetal and infant death. In fact, a very high percentage (68%) of SIUD/SIDS victims with EP developmental alterations had a smoker mother. The work by Bajanowski *et al*. [43] supports this hypothesis. These authors reported a high concentration of cotinine, the major oxidative metabolite of nicotine, in the CSF of SIDS victims with smoker mothers. During pregnancy, about 80 to 90% of the inhaled nicotine is absorbed systemically, as assessed using 14C-nicotine [44]. Most of the constituents of tobacco smoke, in particular nicotine and carbon monoxide, are able to pass through the placental-fetal barrier. By chromatography, Malkawi *et al*. [45] detected significant concentrations of cotinine primarily in CSF samples from newborn babies of smoker mothers, indicating that it rapidly permeates through the blood-brain barrier.

In conclusion, we feel able to say that noxious agents passed from the mother to fetus cause damage to the EP before other autonomic nervous system centers. Therefore, the EP, being the nervous system structure primarily showing structural and/or functional abnormalities in these conditions, should be examined in-depth first in victims of sudden fetal or infant death with smoker mothers.

## Abbreviations

CSF: cerebrospinal fluid; EP: ependyma; GFAP: glial fibrillary acidic protein; SIDS: sudden infant death syndrome; SIUD: sudden intrauterine unexplained death; SVZ: subventricular zone; TdT: deoxynucleotidyl transferase; TUNEL: deoxynucleotidyl transferase-mediated dUTP nick end labeling.

## Competing interests

The authors declare that they have no competing interests.

## Authors' contributions

AML planned the study, analyzed the data and wrote the manuscript with collaborative input and extensive discussion with LM. MFC performed the immunohistochemical methods, helped to evaluate of the related results and was the primary contributor to the figure preparation. All Authors read and approved the final manuscript.
